# Coping strategies and psychological outcomes following the 2023 Kahramanmaraş earthquakes: a systematic review with meta-analytic synthesis

**DOI:** 10.3389/fpubh.2026.1776931

**Published:** 2026-03-04

**Authors:** Metin Çınaroğlu, Eda Yılmazer

**Affiliations:** 1 Psychology Department, Faculty of Administrative and Social Science, İstanbul Nişantaşı University, İstanbul, Türkiye; 2 Psychology Department, Faculty of Social Science, Beykoz University, İstanbul, Türkiye

**Keywords:** anxiety, coping strategies, depression, Kahramanmaraş earthquakes, meta-analysis, posttraumatic growth, PTSD, resilience

## Abstract

**Background:**

The 2023 Kahramanmaraş earthquakes caused unprecedented devastation across southern Türkiye, resulting in widespread psychological distress among survivors. Identifying coping strategies linked to better mental health outcomes is essential for guiding post-disaster support.

**Objective:**

To systematically synthesize quantitative evidence on associations between coping strategies and psychological outcomes (PTSD/trauma symptoms, depression, anxiety, and posttraumatic growth [PTG]) among adult survivors of the 2023 earthquakes, and to meta-analyze associations reported in at least two independent studies.

**Methods:**

Following PRISMA 2020 guidelines and a preregistered OSF protocol (osf.io/7z2pe), four databases (Web of Science, Scopus, PubMed, and DergiPark) were searched for quantitative studies published in Turkish or English. Ten cross-sectional studies (total *N* = 6,223) met inclusion criteria. Data were extracted using a standardized form, study quality was appraised with an adapted Newcastle–Ottawa Scale, and random-effects meta-analyses were conducted for coping–outcome pairs reported in ≥2 studies; remaining associations were summarized descriptively.

**Results:**

Meta-analytic evidence indicated that resilience was consistently associated with lower psychological distress, including post-earthquake trauma severity (pooled r = −0.44), depression (r = −0.41), anxiety (r = −0.43), and overall distress across four studies (r = −0.42). Perceived social support/support-seeking coping was moderately associated with lower PTSD/trauma-related symptoms (pooled r = −0.31). Religious coping (pooled r = −0.21) and positive reappraisal (pooled r = −0.19) showed small inverse associations with PTSD symptoms but with substantial heterogeneity. In single-study analyses, meaning-centered coping and self-compassion were associated with higher PTG, suggesting potentially important pathways that require replication.

**Conclusion:**

Coping processes are meaningfully linked to psychological adjustment after the Kahramanmaraş earthquakes. The most robust evidence supports resilience and social support as protective correlates of lower distress. Evidence for religious coping and positive reappraisal is suggestive but heterogeneous, and PTG-related findings remain preliminary due to limited replication. Longitudinal and intervention-based research is needed to clarify causal mechanisms and inform culturally responsive disaster mental health programs.

**Systematic review registration:**

https://osf.io/7z2pe/overview.

## Introduction

Natural disasters such as earthquakes often inflict severe psychological trauma on affected populations (1). Around the world, earthquake survivors exhibit elevated rates of post-traumatic stress disorder (PTSD), depression, anxiety, and other stress-related symptoms in the months and years following the event (2). The twin earthquakes that struck southern Türkiye on February 6, 2023 – centered in Kahramanmaraş with magnitudes 7.7 and 7.6 – illustrate the profound human impact of such disasters. These earthquakes devastated 11 provinces, causing over 50,000 deaths, more than 100,000 injuries, and the displacement of millions of people (3). In the wake of this catastrophe, early assessments indicated a widespread psychological toll: for example, more than half of surveyed survivors met criteria for probable PTSD in one study shortly after the quake [e.g., (4)]. While meta-analyses of past earthquakes have found somewhat lower average PTSD prevalence (on the order of ~15–25% of survivors), the extraordinary scale and devastation of the Kahramanmaraş disaster likely heightened acute distress (5). High levels of depression and anxiety symptoms have also been reported among survivors, consistent with global disaster research showing that earthquakes commonly precipitate a broad spectrum of psychological difficulties (6, 7). At the same time, trauma responses can vary widely: many survivors experience significant psychopathology, yet others show resilience or even positive psychological changes in the aftermath (8). Indeed, a subset of individuals may achieve posttraumatic growth (PTG) – positive personal changes such as strengthened relationships, life appreciation, or spiritual development – through coping with the adversity, although overall PTG levels after earthquakes tend to be modest on average (9, 10). This backdrop underscores the importance of identifying factors that help mitigate trauma and facilitate recovery following major earthquakes.

Among the most critical factors influencing post-disaster psychological outcomes are coping strategies and resources employed by survivors (6). The 2023 Kahramanmaraş earthquakes prompted a surge of research into how survivors cope and which coping mechanisms are associated with better or worse mental health outcomes [e.g., (11, 12)]. Broadly defined, coping refers to the cognitive and behavioral efforts to manage the internal and external demands of a stressful event (13). Adaptive coping strategies are thought to buffer the impact of trauma, enabling individuals to maintain or regain well-being (14). In the context of earthquake trauma, several key coping domains have been highlighted in the literature: perceived social support, psychological resilience, religious/spiritual coping, positive reappraisal, and self-compassion (15). Each of these has been hypothesized to play a protective role in the aftermath of mass trauma.

Perceived social support – the emotional, informational, and practical assistance received from one’s social network – is one of the most robust predictors of psychological resilience after disasters (1). Strong social support has consistently been linked to lower PTSD severity and reduced risk of depression in trauma survivors (16). Earthquake studies indicate that survivors who feel connected to family, friends, and community tend to report less distress, likely because social support provides opportunities to confide, obtain help, and rebuild a sense of safety (5). Conversely, lacking support or social isolation is a well-established risk factor for chronic PTSD (17). We therefore expected that survivors with higher perceived support would exhibit lower levels of PTSD-related symptoms, depression, and anxiety. Psychological resilience – often defined as the capacity to adapt flexibly and “bounce back” after adversity – is closely related, and is likewise associated with better post-traumatic outcomes. The American Psychological Association conceptualizes resilience as a dynamic process of adapting to life’s challenges through mental, emotional, and behavioral flexibility (18). Empirical studies have shown that greater resilience serves as a protective mechanism against trauma-related disorders (19). For example, survivors high in resilience tend to appraise stressors as more manageable and employ active coping, which can alleviate the severity of PTSD symptoms. We anticipated that in this review, higher resilience would correlate with lower PTSD, anxiety, and depression among earthquake survivors (11).

Coping in the cultural context of Türkiye often includes a significant religious or spiritual dimension, which can be considered an adaptive coping strategy for many individuals (20). Religious coping involves drawing on faith-based beliefs and practices to manage stress (20, 21). Such spiritual coping has been hypothesized to function as a psychological buffer that enhances resilience and aids meaning-making in the face of tragedy (22). Prior research has found that positive religious coping is often associated with lower levels of post-traumatic stress, depression, and fear, as well as with greater posttraumatic growth in highly religious populations (23). By contrast, negative religious coping may impede recovery, though the present review focuses on positive forms. Given this framework, we expected that survivors who engage in religious or spiritual coping would, on average, report more favorable psychological outcomes, including lower trauma-related distress and, in some cases, higher posttraumatic growth (24).

Positive reappraisal (or positive cognitive reframing) is another coping strategy of interest, involving efforts to find positive meaning or growth in the experience of trauma (23). This can include focusing on lessons learned, perceiving oneself as stronger, or viewing the event as an opportunity for spiritual or personal development. Positive reappraisal is considered a facet of meaning-focused coping that can transform one’s appraisal of a stressor. In trauma research, the ability to find meaning in disaster has been linked to lower psychological distress and higher PTG (24). Earthquake survivors who successfully reframe their experience – for example, feeling that surviving the quake gave them a new appreciation for life or brought their community closer – may be less prone to PTSD and more likely to experience growth (25). We hypothesized that higher positive reappraisal (and related meaning-making coping) would correlate with lower trauma-related distress, including PTSD, anxiety, and depression, as well as enhanced posttraumatic growth.

Finally, self-compassion has emerged as a potentially important coping resource in trauma contexts (26). Self-compassion entails treating oneself with kindness, understanding, and forgiveness in times of suffering, rather than with self-criticism or blame (27). It encompasses recognizing that pain and failure are part of the common human experience and responding to one’s own distress with mindfulness and compassion. Although studied less frequently than other coping constructs in disaster settings, self-compassion is theorized to help trauma survivors by counteracting shame and encouraging adaptive emotion regulation (Neff, 2011). Recent evidence suggests that higher self-compassion is associated with lower PTSD symptom severity and greater posttraumatic growth in trauma survivors (28). For example, in a study of Turkish earthquake survivors one year post-disaster, those with more self-compassion reported significantly fewer PTSD symptoms and higher PTG, and self-compassion mediated the relationship between PTSD and growth (29). We anticipated a similar pattern in the present review – namely, that survivors scoring high on self-compassion would exhibit more favorable psychological adjustment, including lower distress and greater positive outcomes.

Existing literature suggests that these adaptive coping strategies – social support, resilience, religious coping, positive reappraisal, and self-compassion – should be protective correlates of mental health following the 2023 earthquakes (6). Building on this theoretical and empirical backdrop, the current study set out to systematically review the quantitative evidence on coping strategies following the 2023 Kahramanmaraş earthquakes and to conduct meta-analyses where sufficient data were available. Accordingly, the present review aimed to assess the magnitude and consistency of associations between coping strategies and psychological outcomes following the 2023 Kahramanmaraş earthquakes, with particular emphasis on resilience and perceived social support. In addition, the review explored associations involving less frequently studied coping processes—such as religious coping, positive reappraisal, and self-compassion—particularly in relation to posttraumatic growth (30). By aggregating data from studies conducted in the aftermath of the Kahramanmaraş earthquakes, this review aims to clarify the magnitude and consistency of observed coping–outcome associations. The ultimate goal is to develop a comprehensive understanding of how coping processes relate to trauma-related outcomes in this context, thereby informing theoretical models of disaster psychology and guiding practical interventions to support survivors’ mental health.

## Methods

### Study design

This systematic review was conducted in accordance with the Preferred Reporting Items for Systematic Reviews and Meta-Analyses (PRISMA) 2020 guidelines. The review protocol was prospectively registered on the Open Science Framework (https://osf.io/7z2pe/overview). The study aimed to synthesize the empirical quantitative evidence on coping strategies among survivors of the 2023 Kahramanmaraş earthquakes and their associations with trauma-related psychological outcomes, including PTSD-related symptoms, depression, anxiety, and posttraumatic growth (PTG). The review employed meta-analytic techniques to estimate pooled effect sizes for coping–outcome associations reported in at least two independent studies, while narrative synthesis was used for associations that were not amenable to quantitative pooling due to limited replication or heterogeneity in measures. Moderator and subgroup analyses were conducted where data were sufficient, and the methodological quality of included studies was appraised using a standardized framework. All stages of the review process—from search and screening to data extraction, quality assessment, and analysis—were performed independently by the authors and in accordance with the preregistered protocol.

### Search strategy

A comprehensive systematic search was conducted to identify empirical studies examining coping strategies and psychological outcomes among survivors of the 2023 Kahramanmaraş earthquakes. The search was performed across four major databases: Web of Science, Scopus, PubMed, and DergiPark (TR Dizin), to capture both international and regionally published literature. Searches were limited to articles published in English and Turkish, from February 6, 2023 (the date of the earthquake) to October 30, 2025. No additional eligible studies were identified between this date and manuscript submission (December 28, 2025). The search strategy combined keywords and Boolean operators covering three core domains: (1) disaster exposure (e.g., “earthquake,” “Kahramanmaraş,” “Türkiye earthquakes”), (2) psychological outcomes (e.g., “PTSD,” “depression,” “anxiety,” “trauma,” “posttraumatic growth”), and (3) coping mechanisms (e.g., “coping strategies,” “resilience,” “social support,” “reappraisal,” “religious coping,” “self-compassion,” “emotion regulation”). Truncations and synonyms were used to maximize sensitivity. An example search string for PubMed was: (“earthquake*” OR “Kahramanmaraş”) AND (“PTSD” OR “posttraumatic stress” OR “depression” OR “anxiety” OR “posttraumatic growth” OR “trauma”) AND (“coping” OR “resilience” OR “social support” OR “reappraisal” OR “religious coping” OR “emotion regulation” OR “self-compassion”) Grey literature and non-indexed Turkish journals were accessed through Dergipark to ensure inclusion of regional research. Reference lists of relevant articles were also hand-searched to identify additional eligible studies. The search and screening process were independently conducted by two reviewers: MÇ and EY (Assistant Professors in Clinical Psychology). Discrepancies were resolved through group discussion and consensus.

### Study selection

All identified records were imported into a reference management system and screened in accordance with the PRISMA 2020 guidelines. Duplicate entries were removed prior to screening. The selection process involved two stages: title and abstract screening, followed by full-text review. In the first stage, one author (EY) and a trained research assistant independently screened titles and abstracts of all retrieved articles to assess potential eligibility based on relevance to the 2023 Kahramanmaraş earthquakes and their focus on psychological coping and trauma-related outcomes. The research assistant received prior training on the eligibility criteria and PRISMA-based screening procedures before screening commenced.

In the second stage, full texts of potentially eligible articles were reviewed in detail by MÇ to confirm inclusion. In cases of uncertainty or ambiguity, full-text eligibility decisions were discussed with the co-author (EY), and final inclusion decisions were reached through consensus. Inclusion decisions were based on pre-established eligibility criteria (see below). Only studies reporting original quantitative data on coping mechanisms and psychological responses among survivors of the Kahramanmaraş earthquakes were retained. Studies that met all inclusion criteria were moved forward for data extraction and quality appraisal. Ultimately, 10 studies met the full inclusion criteria and were included in the final synthesis, with meta-analytic pooling conducted where sufficient data were available.

**Figure 1:**
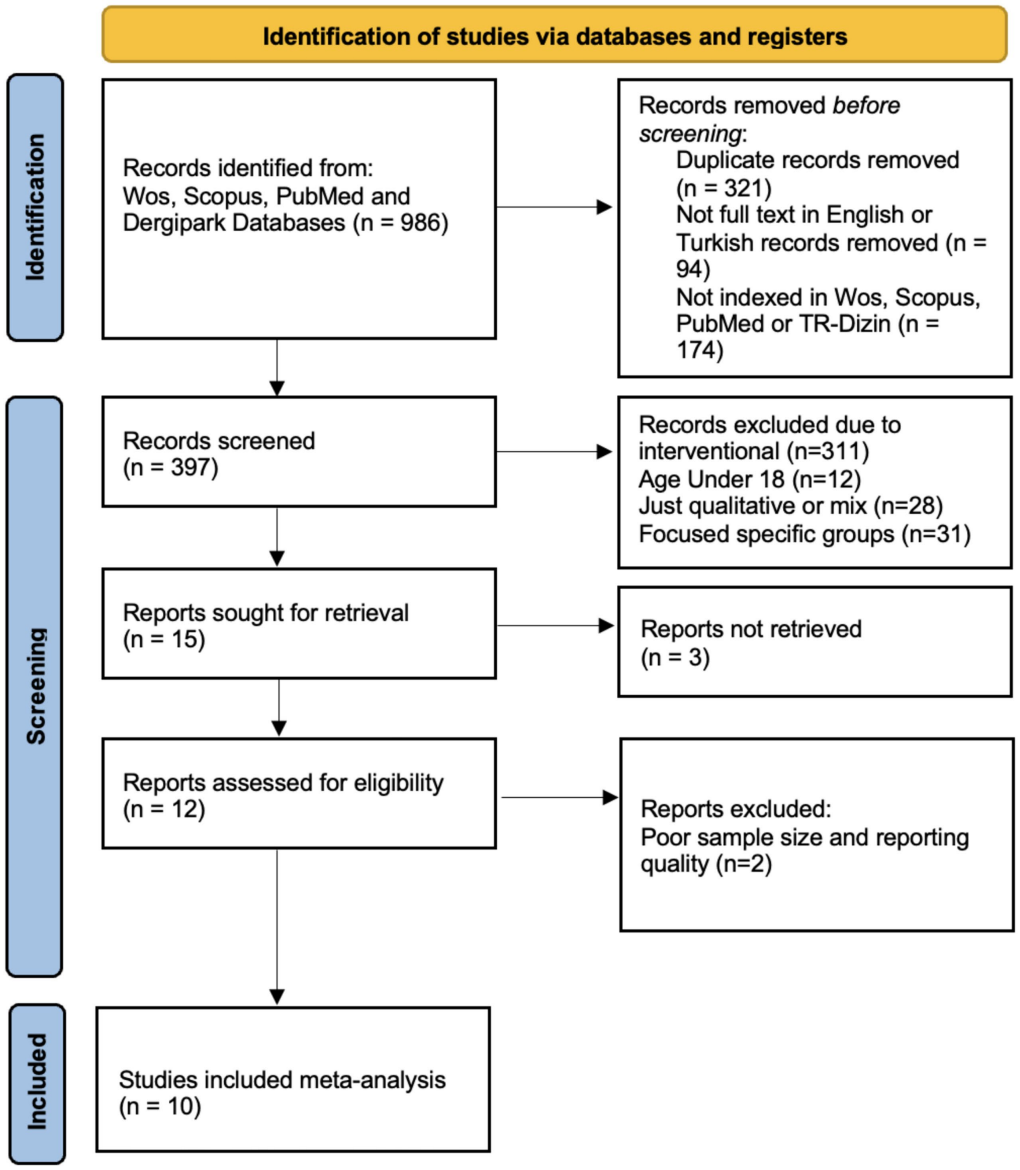
PRISMA flow diagram.

The full study selection process is illustrated in the PRISMA flow diagram (Figure 1).

### Data extraction

A standardized data extraction form was developed to ensure consistency across all included studies. MÇ and research assistant independently extracted data from each study, while the extracted information was reviewed and verified by the other author (EY) for accuracy and completeness. Any discrepancies or ambiguities were resolved through group discussion until full consensus was achieved. For each study, the following information was systematically recorded: study identification details (including authors, publication year, journal, and DOI), sample characteristics (including sample size, mean or range of participant ages, gender distribution, recruitment method, and geographical location), and study design features such as the timing of data collection and whether a cross-sectional survey design was used. In addition, detailed information was collected regarding the psychological constructs assessed, including the specific coping strategies measured (such as perceived social support, religious coping, positive reappraisal, or resilience), the instruments used to assess these constructs, the number of items or subscales, and internal consistency reliability values (e.g., Cronbach’s alpha).

Psychological outcome measures—including PTSD-related symptoms, depression, anxiety, trauma severity, posttraumatic growth (PTG), life satisfaction, and hope—were also documented alongside their respective instruments and psychometric properties. Effect sizes reported in each study were extracted in the form of Pearson’s correlation coefficients (r), standardized regression coefficients (*β*), odds ratios (OR), or group means and standard deviations, depending on availability. Where effect sizes were not directly reported, they were computed or approximated from available statistical data using accepted conversion formulas. If multiple effect sizes were reported for the same predictor–outcome relationship, one primary or most conservative effect size was selected for quantitative synthesis to maintain statistical independence. Information related to statistical modeling approaches—including regression, mediation, and moderation analyses—was recorded where relevant. Particular attention was given to identifying whether coping strategies served as mediators or moderators in the relationship between trauma exposure and psychological outcomes. Additionally, study-level risk-of-bias indicators, such as sample representativeness, use of validated measures, and control of potential confounders, were noted to inform quality assessment. All extracted data were entered into a structured spreadsheet and double-checked prior to synthesis. This comprehensive extraction process ensured uniformity and completeness across all included studies and provided a robust basis for both narrative synthesis and meta-analytic pooling where applicable.

### Quality and bias assessment

The methodological quality and risk of bias of the included studies were assessed using a modified version of the Newcastle–Ottawa Scale (NOS) adapted for cross-sectional studies. This framework evaluates studies across three domains: sample representativeness, measurement reliability and validity, and control of confounding factors. Each study was evaluated by one author (EY) and a research assistant, with disagreements resolved through discussion and consensus. Studies were not excluded based on quality; rather, quality ratings informed interpretation of the findings.

Overall, most studies were rated as moderate quality. Common methodological limitations included reliance on convenience sampling, self-report measures, and cross-sectional designs. Although all studies used validated psychometric instruments with acceptable internal consistency (typically Cronbach’s *α* ≥ 0.80), these features may introduce risks related to selection bias, common method variance, and limited causal inference. Additional sources of potential bias, including limited adjustment for confounding variables, are discussed in detail in the Limitations section. Despite these issues, reporting quality was generally adequate, and the findings were considered suitable for narrative synthesis and meta-analytic pooling, with appropriate caution.

### Statistical analyses

All quantitative analyses were conducted using SPSS (version 30) and Comprehensive Meta-Analysis (CMA, version 3). Meta-analytic pooling employed random-effects models to account for heterogeneity in study populations, coping constructs, and outcome measures. Pearson’s correlation coefficients (r) were used as the primary effect size metric, as they were the most commonly reported statistic across included studies. When necessary, other effect size formats (e.g., standardized regression coefficients or odds ratios) were converted to r using standard transformation formulas to allow for comparability.

Meta-analyses were conducted only for coping–outcome associations reported in at least two independent studies, with one effect size per study included to maintain statistical independence. Separate pooled analyses were performed for eligible coping domains (e.g., resilience, perceived social support, religious coping, positive reappraisal) in relation to trauma-related psychological outcomes (e.g., PTSD-related symptoms, depression, anxiety). Effect sizes were weighted by the inverse of their variance, and 95% confidence intervals were calculated. Statistical heterogeneity was assessed using Cochran’s Q statistic and the I^2^ index, with I^2^ values of approximately 25, 50, and 75% interpreted as low, moderate, and high heterogeneity, respectively.

Formal sensitivity analyses were not conducted, as most pooled estimates were based on a limited number of studies; however, heterogeneity estimates and study-level effect sizes were examined to support cautious interpretation. Moderator analyses were conducted descriptively where two or more studies reported results stratified by variables such as gender, marital status, or injury status; due to limited data, meta-regression was not performed. Narrative synthesis was used to integrate mediation and moderation findings that could not be meta-analyzed because of inconsistent reporting or model structure.

Assessment of publication bias was not formally conducted, as funnel plots and statistical tests for small-study effects are not recommended when fewer than 10 studies contribute to a pooled estimate. Statistical significance was evaluated at *p* < 0.05, and effect sizes were interpreted using conventional benchmarks (r ≈ 0.10 small, r ≈ 0.30 moderate, r ≈ 0.50 large). Findings from pooled analyses and single-study quantitative results were interpreted separately and integrated cautiously in the overall synthesis.

## Results

### Study characteristics of included studies

Ten studies published between 2024 and 2025 met the inclusion criteria, comprising a combined sample of approximately 6,223 earthquake survivors from Türkiye. All studies employed cross-sectional designs and collected data within one year of the February 6, 2023 Kahramanmaraş earthquakes. Sample sizes ranged from 255 to 1,877. Most studies surveyed adults from the general survivor population (mean ages typically 24–42 years), with several samples showing a predominance of female participants [e.g., 61% in Yıldırım et al. (31); 78% in Çağış and Akçe (32)]. Some studies targeted specific subgroups, such as survivors who had migrated away from the affected region (33) or individuals living in temporary shelter areas (34). All data were collected via self-report questionnaires administered either online or in person.

Across the ten studies, a range of coping strategies and psychosocial resources were assessed. Commonly examined domains included perceived social support, religious coping, positive reappraisal, resilience, emotion regulation, life engagement, sense of coherence, psychological flexibility, self-compassion, and social comparison tendencies. Several studies used the Earthquake Stress Coping Scale (92), which measures religious coping, positive reappraisal, and seeking social support. Perceived social support was evaluated in two studies (31, 35), while resilience was assessed in four studies, most commonly via the Brief Resilience Scale. Additional constructs included meaning-centered coping (36), psychological flexibility (37), life engagement (38), emotion regulation difficulties (34), and self-compassion (32). All coping-related instruments demonstrated acceptable reliability (Cronbach’s *α* generally ≥ 0.80).

Psychological outcomes assessed across studies included PTSD-related symptoms, depression, anxiety, trauma severity or exposure indicators, posttraumatic growth (PTG), life satisfaction, and hope. PTSD and trauma-related distress were the most frequently examined outcomes, measured using validated earthquake-specific scales or standard symptom inventories. Depression and anxiety were typically evaluated using instruments such as the PHQ-9, GAD-7, or DASS-21. Several studies also assessed positive psychological outcomes—such as PTG (32, 36), state hope (35), and life satisfaction (39)—to capture adaptive responses. Although terminology varied across studies (e.g., trauma severity, trauma level, post-earthquake experiences), all outcomes reflected either adverse psychological impact or adaptive processes following the disaster. Where available, pooled meta-analytic estimates are presented as the primary quantitative findings, with individual study results summarized concisely to provide context (see Table 1).

**Table 1:** Characteristics of included studies.

Study (year)	Sample (*N*)	Key instruments	Coping constructs	Key outcomes
Yıldırım et al. (2025) (31)	504 young adults (18–30 y; 61% female) from earthquake zone (online survey)	Earthquake anxiety scale; post-earthquake traumatic experiences scale; brief perceived social support questionnaire; brief resilience scale	Perceived social support; resilience	Trauma-related symptoms (traumatic experience severity); earthquake-related anxiety
Yildirim (2024) (35)	323 adults (mean age ≈ 34 y; mixed gender), 4 months post-earthquake	Sense of coherence scale; multidimensional perceived social support scale; state hope scale	Perceived social support; sense of coherence	State hope
Türk et al. (2025) (36)	255 adults (70% female; M_age = 23 y) exposed to the earthquake	Satisfaction with life scale; PTG and posttraumatic depreciation scales (PTGDI-X SF); brief resilience scale (*α* = 0.84); meaning-centered coping scale	Meaning-centered coping (positive reappraisal); resilience	Posttraumatic growth; posttraumatic depreciation
Turan et al. (2025) (37)	330 adults (M_age = 42 y; 46% female) from earthquake region	Perceived ability to cope with trauma (PACT); positive and negative affect schedule; psychological flexibility questionnaire	Coping efficacy (PACT); psychological flexibility; emotional states	Coping efficacy; positive and negative affect
Peker and Cengiz (2025) (33)	1,877 individuals (M_age ≈ 24 y; 84% single) who migrated from the earthquake area	Intolerance of uncertainty scale; earthquake stress coping strategies scale (religious coping, positive reappraisal, social support); post-earthquake trauma scale	Religious coping; positive reappraisal; seeking social support	Trauma-related symptoms (post-earthquake trauma severity)
Özmaya et al. (2025) (38)	1,406 adults (broad age range; mixed gender) from Kahramanmaraş	Short resilience scale; life engagement test; trauma level determination scale	Life engagement; resilience	Trauma severity (composite); resilience
Güler et al. (2024) (39)	388 survivors (18–69 y; M_age = 34.5 y; ≈50% female) from severely affected regions	Social comparison scale; brief resilience scale; satisfaction with life scale; depression and anxiety scales (HADS/DASS)	Social comparison tendency; resilience	Depression symptoms; anxiety symptoms; life satisfaction
Çağlar (2025) (34)	408 survivors (mixed age and gender) from Pazarcık/Elbistan camps	Posttraumatic cognitive attribution scale; difficulties in emotion regulation scale (DERS-16); earthquake stress coping scale; PTSD checklist (PCL-5, short form)	Emotion regulation difficulties; religious coping; positive reappraisal; seeking social support	PTSD-related symptoms
Çağış and Akçe (2025) (32)	317 survivors (≈78% female; ages 18–52 y; M_age = 24.1 y), 1 year post-earthquake	International trauma questionnaire; posttraumatic growth inventory; self-compassion scale	Self-compassion	PTSD-related symptoms; posttraumatic growth
Aktu and Inak (2025) (40)	415 adults (71% female; M_age = 27 y) affected by the earthquake	Brief resilience scale; depression anxiety stress scales (DASS-21); earthquake coping strategies scale	Resilience; social support; positive reappraisal; religious coping	Psychological distress (depression, anxiety, stress); coping with earthquake stress

### Associations between coping strategies and trauma outcomes

**Figure 2:**
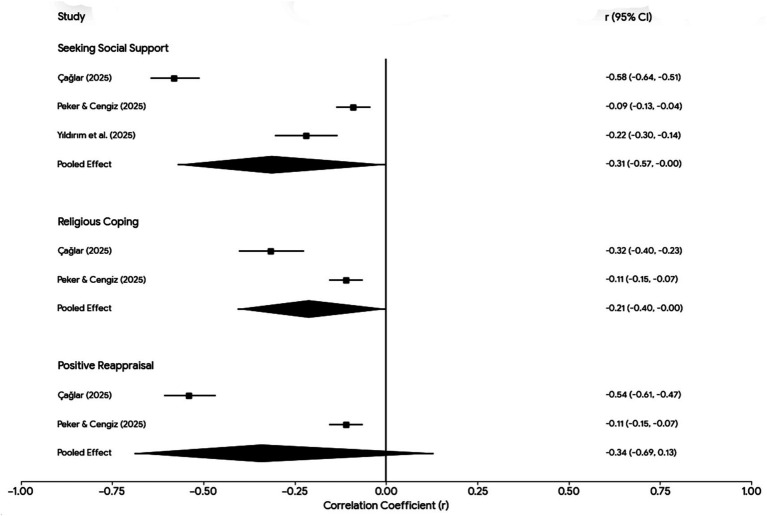
Forest plot of the association between selected coping strategies and trauma-related symptoms.

Across the included studies, higher levels of perceived social support or support-seeking coping were consistently associated with lower trauma-related distress. A random-effects meta-analysis of three studies yielded a pooled effect size of r = −0.31 (95% CI –0.57 to −0.07), indicating a moderate inverse association between social support coping and trauma-related symptoms (Table 2; Figure 2). Study-level effect sizes ranged from r = −0.22 in Yıldırım et al. (31) to r = −0.33 in Çağlar (34), with a smaller association reported in Peker and Cengiz (33) (r = −0.09). In addition to distress-related outcomes, perceived social support was also associated with positive psychological indicators; for example, Yildirim (35) reported a positive correlation between perceived social support and state hope (r = 0.38, *p* < 0.001).

**Table 2:** Meta-analytic pooled associations between coping domains and psychological outcomes (k ≥ 2).

Coping domain	Outcome	Pooled r (95% CI)	I^2^	k (studies)	Included studies
Resilience	Post-earthquake trauma severity/traumatic experiences	−0.44 (−0.55, −0.31)	77%	2	Yıldırım et al. (31) and Özmaya et al. (38)
Resilience	Depression symptoms	−0.41 (−0.47, −0.35)	0%	2	Aktu and Inak (40) and Güler et al. (39)
Resilience	Anxiety symptoms	−0.43 (−0.49, −0.37)	0%	2	Aktu and Inak (40) and Güler et al. (39)
Resilience	General psychological distress*	−0.42 (−0.47, −0.37)	Low	4	Yıldırım et al. (31), Özmaya et al. (38), Aktu and Inak (40), and Güler et al. (39)
Perceived social support/support-seeking coping	PTSD/trauma symptoms	−0.31 (−0.57, −0.07)	72%	3	Yıldırım et al. (31), Peker and Cengiz (33), and Çağlar (34)
Religious coping	PTSD symptoms	−0.21 (−0.40, 0.02)	91%	2	Peker and Cengiz (33) and Çağlar (34)
Positive reappraisal	PTSD symptoms	−0.19 (−0.32, −0.05)	85%	2	Peker and Cengiz (33) and Çağlar (34)

Only associations reported in ≥2 independent studies were meta-analyzed using random-effects models (Fisher’s z transformation). One effect size per study per outcome was included to maintain independence. * General psychological distress includes PTSD/trauma severity, depression, anxiety, or stress indicators. When multiple outcomes were available, one primary distress indicator per study was selected a priori.

Figure 2 displays random-effects meta-analytic estimates for associations between selected coping strategies and trauma-related symptoms following the 2023 Kahramanmaraş earthquakes. Seeking social support showed a statistically significant, moderate inverse association with trauma-related symptoms (pooled r = −0.31, 95% CI [−0.57, −0.07]), although substantial between-study heterogeneity was observed. Effect sizes ranged from a stronger association in Çağlar (34) to weaker associations in Peker and Cengiz (33) and Yıldırım et al. (31).

Religious coping demonstrated a small inverse association with trauma-related symptoms (pooled r = −0.21, 95% CI [−0.40, 0.02]), with considerable heterogeneity across studies, indicating variability in effect magnitude.

Positive reappraisal yielded a pooled estimate with a relatively large mean effect size; however, extreme heterogeneity was present, and the 95% confidence interval crossed zero (pooled r = −0.34, 95% CI [−0.69, 0.13]), indicating that the overall association was not statistically significant. These findings suggest that while support-seeking coping shows the most consistent protective association, effects for religious coping and positive reappraisal vary substantially across samples and should be interpreted with caution.

Table 3 presents the extracted study-level associations between coping strategies and psychological outcomes across the included studies. These effect sizes formed the basis for subsequent meta-analytic pooling and narrative synthesis.

**Table 3:** Extracted effect sizes (correlations r or standardized *β* coefficients) between coping strategies and key outcomes in each included study.

Study	Coping–outcome pair	Effect size (r or β)	Notes
Yıldırım et al. (31)	PSS → Trauma severity (PTSD symptoms) Resilience → Trauma severity	r = −0.22** r = −0.50**	Both PSS and resilience negatively correlated with traumatic stress. In mediation, PSS (β = −0.07*) and resilience (β = −0.19***) independently predicted lower trauma.
Yildirim (35)	Sense of coherence → Hope PSS → Hope	r = +0.45** r = +0.38**	Coherence and social support both positively correlated with state hope. PSS partially mediated coherence’s effect on hope (indirect ~0.07**).
Türk et al. (36)	Meaning-centered coping → PTG Meaning-centered coping → PT Depreciation (PTD)	r = +0.67** r = −0.46**	Large associations: finding meaning strongly increased growth and reduced depreciation. In mediation, meaning-centered coping had a sizable indirect effect on PTG (0.64**) and PTD (−0.26**).
Turan et al. (37)	Psych. flexibility ↔ PACT (coping efficacy) Neg. affect ↔ PACT Pos. affect ↔ PACT	r = +0.888** r = +0.884** r = −0.894**	Extremely high correlations: those with high coping ability had high flexibility and high negative affect (and low positive affect). PF + emotions explained 88% of variance in coping ability.
Peker and Cengiz (33)	IU → PTSD symptoms Social support coping → PTSD Positive reappraisal → PTSD Religious coping → PTSD	r = +0.45** r = −0.09* r = −0.11* r = −0.11*	IU correlated moderately with PTSD (r ~ 0.45). All three coping subscales had small protective correlations (−0.09 to −0.11). Mediation: IU had negative paths to coping (β: −0.05* to −0.03*) and coping had negative paths to PTSD (β: −0.39*** to −0.16*). Total model R^2^ = 0.217.
Özmaya et al. (38)	Resilience → Trauma level Life engagement → Trauma level Resilience → Life engagement	r = −0.38** r = −0.17** r = +0.31**	Resilience and life engagement both inversely correlated with trauma severity. Life engagement also positively linked to resilience. Life engagement significantly mediated resilience’s effect on trauma (indirect point estimate = −0.068, *p* < 0.10).
Güler et al. (39)	Social comparison → Depression Social comparison → Anxiety Social comparison → Life satisfaction	r = +0.23** r = +0.22** r = −0.31**	Higher tendency to compare with others was associated with more depression/anxiety and lower satisfaction. Social comparison also correlated −0.20** with resilience, indicating a broad negative impact.
Çağlar (34)	Posttr. cognitions → Emotion dysregulation Posttr. cognitions → (+) Reappraisal Posttr. cognitions → (+) Religious coping Posttr. cognitions → (+) Social support coping Emotion dysregulation → PTSD Positive reappraisal → PTSD Religious coping → PTSD Social support → PTSD	β = +0.240*** β = −0.382*** β = −0.286*** β = −0.330*** β = +0.654*** β = −0.541*** β = −0.317*** β = −0.581***	All mediation paths significant in hypothesized directions. Maladaptive cognitions increased DERS (emotion-regulation problems) and decreased all coping strategies, which in turn influenced PTSD. Notably, seeking social support showed a large direct effect (β = −0.581) on reducing PTSD symptoms. Overall model R^2^ ~ 0.57 for PTSD.
Çağış and Akçe (32)	PTSD symptoms → Self-compassion PTSD symptoms → PTG Self-compassion → PTG	r = −0.31** r = +0.15** r = +0.22**	PTSD severity was moderately associated with lower self-compassion, and weakly with higher PTG. Self-compassion had a positive link to PTG. Mediation: PTSD had a significant *indirect* effect on PTG via reduced self-compassion (partial mediation, *p* < 0.01).
Aktu and Inak (40)	Resilience → Anxiety Resilience → Stress Resilience → Depression Resilience → Coping (CES) Depression → Coping (CES)	r = −0.44** r = −0.37** r = −0.41** r = +0.18** r = −0.21**	Resilience was strongly inversely correlated with distress (anxiety, stress, depression). Higher resilience modestly improved overall coping (CES). In serial mediation, resilience increased coping both directly (β ~ 0.13*) and indirectly by lowering anxiety/stress, which in turn lowered depression and thus increased coping. (Females had slightly lower CES than males.)

*p < 0.05, **p < 0.01, ***p < 0.001. Effect sizes are Pearson correlations (r) unless otherwise noted (β). “+” denotes a positive relationship; “–” denotes a negative relationship. PTG = posttraumatic growth; PTD = posttraumatic depreciation; PSS = perceived social support; DERS = Difficulties in Emotion Regulation; PTCA = posttraumatic cognitive attributions; CES = coping with earthquake stress (total score).

### Meta-analysis of coping effects on psychological outcomes

**Figure 3:**
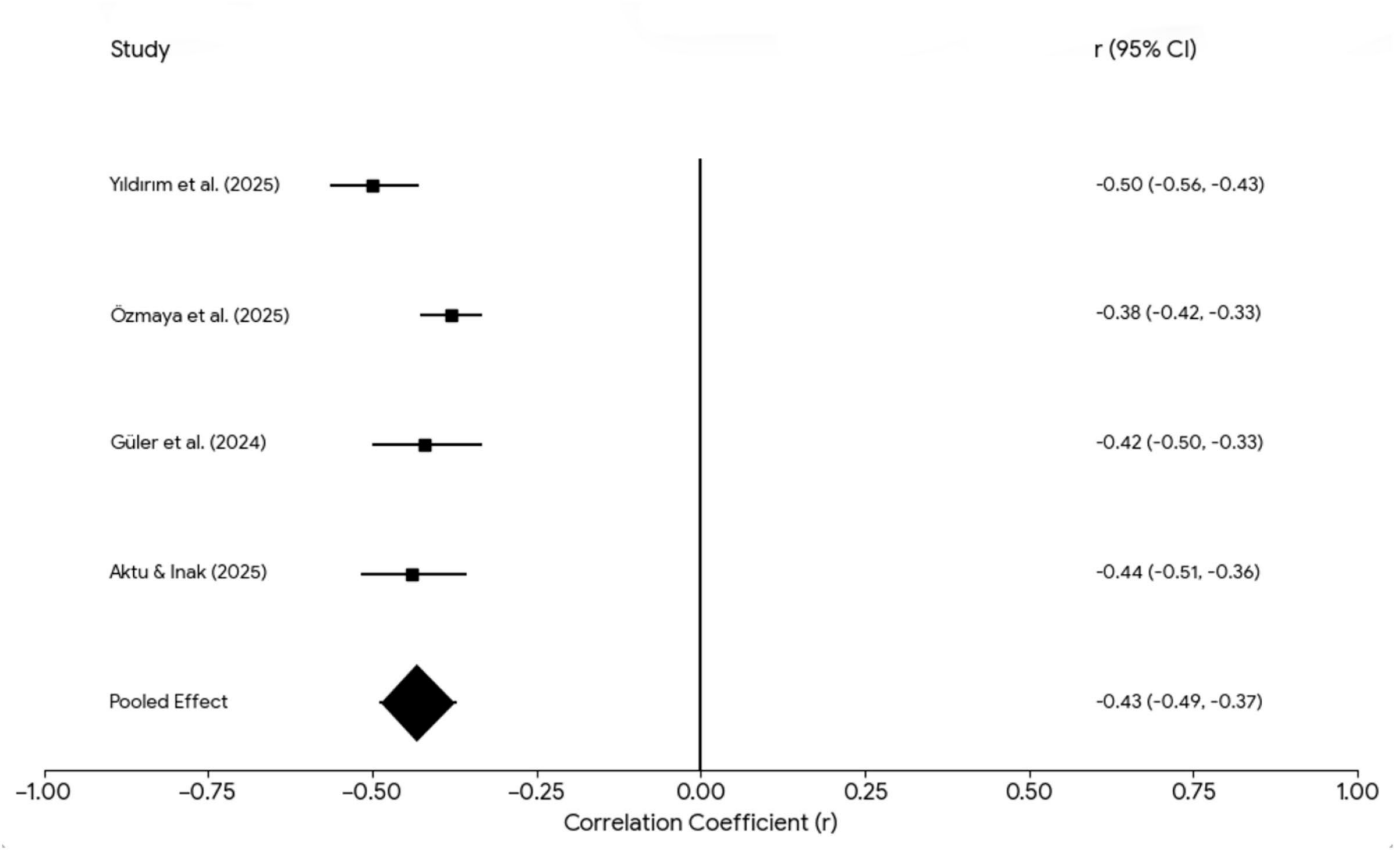
Forest plot of the association between resilience and psychological distress.

A series of random-effects meta-analyses were conducted for coping–outcome associations reported in at least two independent studies (Table 2; Figures 2, 3). Resilience showed the strongest and most consistent associations with psychological distress, with pooled correlations of r = −0.44 (95% CI –0.55 to −0.31) for trauma-related symptoms, r = −0.41 (95% CI –0.47 to −0.35) for depression, and r = −0.43 for anxiety, each based on two studies. Perceived social support/support-seeking coping, examined in three studies, yielded a pooled inverse association with trauma-related symptoms (r = −0.31, 95% CI –0.57 to −0.07).

Religious coping, assessed in two studies, demonstrated a small inverse pooled association (r = −0.21, 95% CI –0.40 to 0.02), accompanied by substantial between-study heterogeneity. Positive reappraisal, also assessed in two studies, showed a small pooled inverse association (r = −0.19, 95% CI –0.32 to −0.05) with high heterogeneity. Overall, the meta-analytic findings indicate that adaptive coping strategies are generally associated with lower trauma-related psychological distress, although the magnitude and consistency of effects varied across coping domains.

Figure 3 presents a random-effects forest plot of the association between resilience and psychological distress (including PTSD-related symptoms, depression, or anxiety) following the 2023 Kahramanmaraş earthquakes. Pooling data from four independent studies (total *N* = 2,713), higher resilience was consistently associated with lower levels of psychological distress, yielding a robust inverse association (pooled r = −0.42, 95% CI [−0.47, −0.37]). Individual study effect sizes were relatively homogeneous, ranging from r = −0.38 (38) to r = −0.50 (31), indicating low between-study variability and supporting resilience as a consistent protective correlate of post-earthquake psychological distress.

### Religious coping

Across studies, higher levels of religious coping were generally associated with lower trauma-related symptoms. Peker and Cengiz (33) reported a small inverse correlation (r = −0.11, *p* < 0.05), whereas Çağlar (34) observed a stronger association with PTSD-related symptoms (r = −0.29, *p* < 0.001). In Çağlar’s mediation model, posttraumatic cognitions predicted lower religious coping (*β* = −0.286), which in turn predicted higher PTSD-related symptoms (*β* = −0.138). A random-effects meta-analysis of these two studies yielded a pooled correlation of r = −0.21 (95% CI –0.40 to 0.02), indicating a small and heterogeneous association that did not reach conventional statistical significance (Table 2; Figure 2).

### Positive reappraisal and meaning-making

Positive cognitive reappraisal showed inverse associations with trauma-related symptoms across studies. Peker and Cengiz (33) reported a small negative correlation (r = −0.11, *p* < 0.01), whereas Çağlar (34) identified a larger association (r = −0.38, *p* < 0.01). In Çağlar’s model, positive reappraisal was negatively associated with posttraumatic cognitions (*β* = −0.541) and inversely associated with PTSD-related symptoms (β = −0.206). Meta-analytic pooling of the two available studies yielded a small inverse association (r = −0.19, 95% CI –0.32 to −0.05), accompanied by substantial heterogeneity (Table 2; Figure 2). In addition, a single study examining meaning-centered coping reported strong positive associations with posttraumatic growth (r = 0.67, *p* < 0.01) and inverse associations with posttraumatic depreciation (r = −0.43, *p* < 0.01) (36) (Table 4).

**Table 4:** Quantitative findings not meta-analyzed (single-study evidence, k = 1).

Coping domain	Outcome	Effect Size	Study
Meaning-centered coping	Posttraumatic growth (PTG)	r = +0.67	Türk et al. (36)
Meaning-centered coping	Posttraumatic depreciation	r = −0.46	Türk et al. (36)
Self-compassion	PTSD symptom severity	r = −0.31	Çağış and Akçe (32)
Self-compassion	Posttraumatic growth	r = +0.22	Çağış and Akçe (32)
Emotion-regulation difficulty	PTSD symptom severity	r ≈ +0.24	Çağlar (34)
Psychological flexibility	Coping efficacy (PACT)	r ≈ +0.89	Turan et al. (37)
Social comparison tendency	Depression	r = +0.23	Güler et al. (39)
Social comparison tendency	Anxiety	r = +0.22	Güler et al. (39)

The following associations were reported in only one eligible study each and therefore were not pooled. They are presented as quantitative narrative synthesis to inform hypothesis generation.

### Emotion regulation and psychological flexibility

Emotion-regulation difficulties were positively associated with trauma-related symptom severity. In Çağlar (34), difficulties in emotion regulation correlated with higher PTSD-related symptoms (r ≈ 0.24, *p* < 0.001) and mediated the association between posttraumatic cognitions and PTSD-related symptoms (β cognition→DERS = 0.240; β DERS→PTSD = 0.474).

Turan et al. (37) reported strong associations between perceived ability to cope with trauma (PACT) and psychological flexibility (r = 0.888), negative affect (r = 0.884), and positive affect (r = −0.894). Psychological flexibility and emotional states collectively explained 88.3% of the variance in coping ability, with both positive affect (β = −0.309) and negative affect (β = 0.331) emerging as significant predictors.

### Life engagement, sense of coherence, and hope

Life engagement was significantly associated with trauma outcomes. Özmaya et al. (38) found that life engagement correlated negatively with trauma severity (r = −0.17, *p* < 0.01) and positively with resilience (r = 0.31). Life engagement partially mediated the association between resilience and trauma severity (β resilience→engagement = 0.31; β engagement→trauma = −0.22).

Yildirim (35) reported positive correlations between sense of coherence and state hope (r = 0.45, *p* < 0.001), and between perceived social support and hope (r = 0.38, *p* < 0.001). Mediation analysis indicated that perceived social support partially explained the association between coherence and hope (indirect effect ≈ 0.07).

### Self-compassion

Çağış and Akçe (32) examined self-compassion as a mediator between PTSD-related symptoms and posttraumatic growth one year after the earthquake. PTSD-related symptoms were negatively correlated with self-compassion (r = −0.31, *p* < 0.01) and positively correlated with posttraumatic growth (r = 0.15, *p* < 0.01). Self-compassion was positively associated with posttraumatic growth (r = 0.22, *p* < 0.01), and mediation analysis indicated a significant indirect effect of PTSD-related symptoms on posttraumatic growth through self-compassion (unstandardized indirect ≈ − 0.07, *p* < 0.01) (Table 4).

### Social comparison

Güler et al. (39) found that social comparison tendencies were associated with poorer psychological outcomes. Social comparison correlated positively with depression (r = 0.23, *p* < 0.001) and anxiety (r = 0.22, *p* < 0.001), and negatively with life satisfaction (r = −0.31, *p* < 0.001) and resilience (r = −0.20, *p* < 0.01), indicating that greater engagement in social comparison was linked to higher distress and lower well-being (Table 4).

Table 2 summarizes the results of random-effects meta-analyses examining associations between coping strategies and psychological outcomes reported in at least two independent studies. Resilience demonstrated consistent and moderate-to-large inverse associations with post-earthquake trauma severity (pooled r = −0.44), depressive symptoms (r = −0.41), anxiety symptoms (r = −0.43), and overall psychological distress across four studies (r = −0.42). Perceived social support or support-seeking coping was moderately associated with lower PTSD or trauma-related symptoms (r = −0.31), although heterogeneity was substantial. Religious coping and positive reappraisal showed small inverse associations with PTSD symptoms, with considerable between-study heterogeneity. Overall, pooled effects consistently indicated that higher levels of adaptive coping were associated with lower psychological distress following the earthquakes.

Table 4 presents quantitative findings from individual studies for coping–outcome associations that were reported in only one eligible study and therefore could not be meta-analyzed. These results are presented as descriptive quantitative evidence and should be interpreted cautiously. Single-study analyses suggested that meaning-centered coping and self-compassion were positively associated with posttraumatic growth, while emotion regulation difficulties and social comparison tendencies were associated with higher psychological distress. Although these findings are theoretically informative, the lack of replication precludes quantitative synthesis, and further studies are required to determine the robustness of these associations.

### Subgroup differences and exploratory moderation findings

Several studies explored whether coping–outcome associations differed across demographic or exposure-related subgroups (Table 5). Gender differences were reported in multiple samples: in Peker and Cengiz (33), women showed higher PTSD symptom levels than men (45.6% vs. 24.9% in the high-PTSD cluster; t₍₁₈₇₅₎ = 10.91, *p* < 0.05), and Aktu and Inak (40) found that female gender was associated with lower coping efficacy (*β* = −0.131, *p* < 0.01). No study reported significant gender-based moderation of coping–outcome associations. Age-related moderation was not examined directly. Most samples consisted of young to middle-aged adults, and stratified analyses were not conducted. In Yildirim (35), older (primarily married) participants reported higher hope and coherence than younger participants, although age itself was not tested as a moderator. Marital status was associated with several outcomes. Yildirim (35) reported higher hope (t = 3.70, *p* < 0.001) and coherence (t = 2.43, *p* < 0.05) among married survivors compared to single individuals, and Aktu and Inak (40) found that marriage predicted higher coping scores (β = +0.115, *p* < 0.05). Exposure severity showed consistent associations with psychological outcomes. Yıldırım et al. (31) observed that survivors with physical injuries reported higher anxiety (M = 16.43 vs. 13.84) and trauma scores (M = 67.1 vs. 53.2), both *p* < 0.001, compared with non-injured participants. Levels of perceived social support and resilience did not differ significantly by injury status. Comparisons of coping strategy effects across studies indicated variability in the magnitude of associations. In Peker and Cengiz (33), indirect effects for positive reappraisal (β_indirect = −0.043) and religious coping (β_indirect = −0.039) were larger than for social support (β_indirect = −0.016). In Çağlar (34), the standardized effect of social support on PTSD (β = −0.386) exceeded that of religious coping (β = −0.138). No study formally tested interaction terms such as gender × coping or age × coping.

**Table 5:** Subgroup differences and exploratory moderation-related findings reported by the included studies.

Moderator	Study (year)	Finding
Gender	Peker and Cengiz (33)	Women had significantly higher PTSD symptom scores than men (45% vs. 25% in high-PTSD category). No gender difference in coping usage reported.
	Aktu and Inak (40)	Female gender associated with slightly lower coping-with-stress score (β = −0.13, *p* < 0.01).
Age	Yildirim (35)	Older (married) participants showed higher hope and coherence than younger (single) participants. No direct age moderation tested.
	(No other age analyses)	Earthquake impact was largely similar across age groups in reported studies.
Marital status	Yildirim (35)	Married survivors had higher hope (*p* < 0.001) and coherence (*p* < 0.05) than singles.
	Aktu and Inak (40)	Being married predicted higher coping levels (β = +0.115, *p* < 0.05).
Physical injury	Yıldırım et al. (31)	Injured survivors reported greater earthquake anxiety and trauma symptoms than non-injured (mean PTSD score 67.1 vs. 53.2, *p* < 0.001). PSS and resilience did not differ by injury status.
Coping type	Peker and Cengiz (33)	Positive reappraisal and religious coping showed larger mediating effects on PTSD than social support in this sample.
	Çağlar (34)	Seeking social support had a stronger direct effect on PTSD (β = −0.39) than religious coping (−0.14). Emotion regulation difficulty was the strongest risk mediator (β = +0.47).
Time since quake	(Implicit comparisons)	Studies conducted later (1 year post-quake) still found similar coping-outcome relationships (e.g., self-compassion→PTG), suggesting coping effects persist over time. No longitudinal moderation tested.

Table 5 summarizes subgroup differences and exploratory moderation-related findings reported in the included studies. Across studies, several demographic and exposure-related characteristics—such as gender, marital status, age, and physical injury—were associated with variation in psychological outcomes or coping levels. However, these findings were derived from study-specific comparisons rather than formal interaction tests or meta-analytic moderation analyses. No study conducted statistical tests of coping × subgroup interactions, and therefore the results presented in Table 5 should be interpreted as descriptive and exploratory.

### Risk of bias and study quality

All included studies used observational, cross-sectional designs, with coping and outcome measures collected at the same time point. As a result, none of the studies permitted temporal or causal inference (Table 6). All studies relied exclusively on self-report instruments, introducing potential for common-method bias. Across studies, established coping and psychological outcome measures were used, with internal consistency coefficients typically ranging from *α* = 0.80 to 0.95. Sample sizes were generally adequate: seven studies enrolled more than 400 participants, and two studies (33, 38) exceeded 1,400 participants.

**Table 6:** Quality appraisal and potential sources of bias in the included studies.

Study	Design and sampling	Potential biases	Quality rating
Yıldırım et al. (31)	Cross-sectional; convenience online sample of young adults from quake region.	Self-selection bias (young, internet-users overrepresented). Single-time self-report. No control for confounders.	⭐⭐⭐☆ Moderate – good measures, large *N* = 504, but cross-sectional.
Yildirim (35)	Cross-sectional; convenience sample via university network (4 months post-quake).	Sample relatively small (*N* = 323) and locale-specific (Adana). Possible social desirability in reporting hope.	⭐⭐⭐☆ Moderate – some risk of sampling bias, but analytic rigor good.
Türk et al. (36)	Cross-sectional survey; recruited survivors (likely students and community) online.	Smaller sample (*N* = 255) – may limit generalizability. Cross-sectional; cannot infer causal direction (life satisfaction vs. PTG).	⭐⭐⭐☆ Moderate – well-conducted with validated scales, but underpowered for some analyses.
Turan et al. (37)	Cross-sectional; community sample (mixed ages, urban Turkey). Data collection not specified (likely paper survey).	Potential recall bias (self-report of coping ability). High correlations suggest possible common-method inflation.	⭐⭐⭐☆ Moderate – results plausible but extremely high r’s warrant caution.
Peker and Cengiz (33)	Cross-sectional; very large sample (*N* = 1,877) of migrants from quake zone, recruited via multi-center effort.	Strong sample size but 84% single and relatively young – not fully representative. Cross-sectional mediation (direction IU → coping assumed, not proven).	⭐⭐⭐☆ Moderate – large *N* improves reliability; some confidence in mediation but still causal ambiguity.
Özmaya et al. (38)	Cross-sectional; large volunteer sample (*N* = 1,406) from Kahramanmaraş, online survey.	Possibly overrepresents more educated survivors (online form). Trauma and resilience self-reported; no clinical verification.	⭐⭐⭐☆ Moderate – solid sample and stats, minor selection bias likely.
Güler et al. (39)	Cross-sectional; paper-and-pencil survey of 388 survivors in hard-hit provinces.	Multi-region sample improves generalizability. However, no control for time since trauma or prior mental health. Social comparison measure was non-standard (2 items).	⭐⭐⭐☆ Moderate – novel insights but some measurement limitations.
Çağlar (34)	Cross-sectional; convenience sample (*N* = 408) in tent cities (post-quake shelters).	Possibly a more severely affected sample. Cross-sectional multiple mediation – infers causal chain without temporal data. Self-report PTSD may overlap with “cognitions” predictor (common method bias).	⭐⭐⭐☆ Moderate – appropriate analyses and large effects, but directionality not certain.
Çağış and Akçe (32)	Cross-sectional; volunteer sample (*N* = 317, mostly female) ~ 1 year post-disaster.	Follow-up timing good (1y), but sample is predominantly young females – may bias PTG levels. Controlled for demographics in mediation which strengthens causal inference slightly.	⭐⭐⭐☆ Moderate – results are plausible, some sample bias; no major flaws aside from cross-sectional design.
Aktu and Inak (40)	Cross-sectional; convenience sample of 415 adults, recruited through a university in Siirt.	Measures (DASS, BRS, etc.) well-validated. Included covariates (gender, marital) which adds rigor. Still, serial mediation is based on cross-sectional data – temporal ordering (resilience vs. depression) assumed, not proven.	⭐⭐⭐☆ Moderate – one of the stronger analyses (covariate control), but causality cannot be confirmed.

Quality ratings reflect a modified Newcastle–Ottawa scale adapted for cross-sectional designs; higher star counts indicate lower overall risk of bias.

Sampling bias was present in most studies due to reliance on convenience recruitment, often via online platforms or university networks. Some demographic groups—such as older adults or individuals with limited internet access—were likely underrepresented, and several samples showed substantial gender imbalance. Measurement timing varied, with some studies collecting data shortly after the earthquakes and others up to one year later; this variation may partly account for the heterogeneity observed in several pooled estimates. Most analyses did not adjust for potential confounding variables, and only a small number of studies included demographic covariates in regression or mediation models.

No major reporting inconsistencies were identified; study aims, methods, and outcomes were generally described clearly and transparently. Using a modified Newcastle–Ottawa Scale adapted for cross-sectional designs, most studies were rated as moderate quality. Evidence of publication bias could not be formally assessed due to the limited number of studies contributing to most pooled analyses; however, the inclusion of large samples, smaller local journals, and studies reporting non-significant subgroup findings suggests reasonably broad coverage of the available evidence.

## Discussion

This systematic review with meta-analytic synthesis provides a comprehensive overview of the psychological aftermath of the 2023 Kahramanmaraş earthquakes, highlighting the role of coping strategies in shaping survivor outcomes. Overall, adaptive coping mechanisms were generally associated with more favorable psychological adjustment among survivors. This pattern is consistent with the transactional model of stress and coping, which posits that problem-focused and active coping efforts can attenuate distress in high-stress environments (13, 41).

Among the coping domains examined, resilience emerged as the most robust and consistent protective factor. Higher resilience was associated with lower levels of psychological distress, including PTSD-related symptoms, depression, and anxiety, with pooled effect sizes in the moderate-to-large range. These findings are highly congruent with recent studies conducted in the aftermath of the Kahramanmaraş earthquakes, which have similarly shown that resilience is closely linked to better mental health outcomes and may buffer the impact of trauma exposure (42, 43). More broadly, the central role of resilience observed here aligns with the disaster mental health literature, where resilience is repeatedly identified as a key protective correlate against post-traumatic psychopathology (1, 8). Survivors who are able to flexibly adapt and recover in the face of adversity appear less likely to experience severe or persistent psychological distress, a pattern also reported following other large-scale earthquakes, such as those in Wenchuan and Haiti (44, 45). Taken together, the consistency and magnitude of the observed associations reinforce the view that strengthening psychological resilience may be a particularly important target for post-disaster mental health interventions (46).

Perceived social support was the second coping domain demonstrating a comparatively strong and reliable protective association. Meta-analytic results indicated that survivors reporting higher levels of perceived social support or support-seeking coping tended to exhibit lower trauma-related psychological symptoms on average. This finding is highly consistent with extensive prior research identifying social support as one of the most robust predictors of post-trauma mental health (17, 47). Social support may facilitate recovery by providing emotional reassurance, practical assistance, and a sense of belonging during the disorganizing aftermath of disaster (48). Even in contexts of mass trauma, the availability of family, friends, or community resources can reduce feelings of fear and helplessness and may attenuate the development of PTSD and depressive symptoms (1, 49).

An important theoretical implication of the present synthesis is that perceived social support should not be conceptualized solely as an individual-level coping strategy, but rather as a fundamentally *societal and community-level process*. Although most included studies assessed social support via individual self-report measures, the observed protective associations likely reflect broader social dynamics embedded within families, neighborhoods, religious communities, and informal aid networks. In the context of the Kahramanmaraş earthquakes—where entire communities were simultaneously affected—supportive interactions did not occur in isolation but emerged from collective efforts to share resources, provide shelter, and restore a sense of safety and belonging. This interpretation aligns with disaster recovery models emphasizing social cohesion and collective efficacy as core determinants of psychological resilience following mass trauma. Rather than functioning merely as a personal buffer against stress, social support in disaster settings may operate as a *community-level intervention*, mitigating distress through shared meaning-making, mutual aid, and the re-establishment of social norms. Accordingly, interventions that aim to strengthen post-disaster mental health may benefit from prioritizing community-based approaches—such as peer support groups, neighborhood initiatives, and family-centered interventions—over exclusively individual-focused models (50).

At the same time, some heterogeneity in effect sizes was observed across studies, suggesting that the magnitude of social support’s protective role may vary depending on contextual and methodological factors. Differences in how social support was operationalized (e.g., general perceived support versus earthquake-specific support) (51), as well as sample characteristics and living conditions, may partially account for this variability. Nevertheless, both the present findings and prior meta-analytic evidence consistently underscore the importance of social connectedness as a central component of psychological recovery following disasters (17, 52).

Our review also examined the role of religious and spiritual coping, a culturally salient coping form in this context (53). Meta-analytic findings indicated a small inverse association between religious coping and trauma-related psychological distress, suggesting that survivors who engaged more frequently in religious coping tended, on average, to report slightly lower levels of distress. Although this association was weaker and less consistent than those observed for resilience or perceived social support, it remains meaningful given the strong religious and spiritual orientation of many communities affected by the earthquakes (54). Engagement in faith-based practices—such as prayer, trust in God’s will, and participation in religious communities—may have provided comfort or facilitated meaning-making for some survivors, potentially contributing to psychological relief (55). This interpretation is broadly consistent with prior evidence indicating that positive religious coping can be associated with lower depression and anxiety and improved well-being following natural disasters (56, 57).

At the same time, the modest magnitude and heterogeneity of the observed association suggest important sources of variability. One explanation is the distinction between positive and negative forms of religious coping: while constructive religious responses (e.g., feeling supported or guided by faith) are often linked to better outcomes, maladaptive interpretations—such as viewing the disaster as divine punishment—may exacerbate guilt or distress (58, 59). Few studies in the current review explicitly differentiated between these dimensions, limiting more nuanced conclusions. Individual differences may also play a role; survivors with higher intrinsic religiosity may derive greater psychological benefit from religious coping, whereas those for whom religion is less central may experience little effect (60). Nonetheless, within a sociocultural context where religion constitutes an important source of meaning and support, these findings suggest that spiritually integrated coping approaches may represent a potential, though not universally effective, component of post-disaster recovery. Similar patterns have been reported in other disaster settings, including following the 2010 Haiti earthquake, where spiritual practices were associated with reduced PTSD symptoms and enhanced meaning-making (61, 62).

The present findings also highlight the importance of conceptualizing religious coping as a *bilateral construct*, encompassing both potentially adaptive and maladaptive dimensions. While the pooled analyses indicated a small inverse association between religious coping and trauma-related distress, substantial heterogeneity was observed across studies. This variability suggests that the psychological impact of religious coping may depend critically on its qualitative form. Positive religious coping—such as perceiving spiritual support, engaging in prayer for comfort, or interpreting survival through benevolent meaning—may foster emotional regulation and hope, whereas negative religious coping—such as interpreting the disaster as divine punishment or abandonment—may exacerbate guilt, fear, and psychological distress. Importantly, most studies included in the current review did not systematically distinguish between positive and negative religious coping styles, limiting more nuanced synthesis. From a theoretical perspective, this underscores the need for future meta-analytic work that explicitly differentiates these dimensions and examines their potentially divergent associations with mental health outcomes. Given the cultural salience of religion in Türkiye and similar contexts, testing the bilateral effects of religious coping represents a critical next step for refining trauma-coping models and informing culturally sensitive interventions.

Positive reappraisal and related meaning-making strategies were also linked to more favorable psychological outcomes, although with considerable heterogeneity across studies. On average, individuals who reported greater use of positive reappraisal—such as identifying personal growth, renewed life priorities, or strengthened social bonds following the disaster—tended to report lower trauma-related symptoms and higher indicators of positive adjustment. Evidence from individual studies supports this pattern: for example, one study reported a strong association between meaning-centered coping and posttraumatic growth (r ≈ 0.67), suggesting a potentially important meaning-making pathway that warrants cautious interpretation and further replication (36). Another study found that positive reappraisal was inversely associated with PTSD-related symptoms in the presence of adaptive posttraumatic cognitions (34). However, other studies reported only weak associations (33), resulting in an overall mixed and heterogeneous pattern at the pooled level.

Several factors may help explain the observed variability in findings related to positive reappraisal. The effectiveness of positive reappraisal likely depends on contextual opportunities and resources: survivors who received adequate social support and had sufficient time for reflection may have been better positioned to construct positive meaning, whereas those still facing acute stressors or lacking psychosocial resources may have struggled to reframe their experiences (63). Timing also appears relevant. Studies conducted shortly after the earthquakes—when basic needs were often unmet—tended to show weaker associations between reappraisal and reduced distress, whereas studies conducted several months later, or closer to the one-year mark, more often linked reappraisal and meaning-making to psychological growth and recovery. This pattern is theoretically coherent, as meaning-making processes are thought to emerge more prominently during later phases of adjustment rather than in the immediate aftermath of trauma (64, 65). Cultural factors may further contribute to heterogeneity; in some contexts, meaning-making may be expressed primarily through spiritual narratives (e.g., attributing survival to divine purpose), which may not be fully captured by secular measures of positive reappraisal (53). Taken together, these findings provide tentative support for the adaptive potential of positive reappraisal following mass trauma, consistent with theoretical models of posttraumatic growth that emphasize meaning-making (10, 66), while also highlighting substantial individual and contextual variability. Future qualitative and longitudinal studies may help clarify how survivors construct meaning over time and which conditions facilitate adaptive reappraisal processes.

Although examined in fewer studies, self-compassion showed promising associations with psychological outcomes. In one study included in the review, higher self-compassion was moderately associated with lower PTSD-related symptoms and positively associated with greater posttraumatic growth, and mediation analyses suggested that self-compassion partially explained the link between trauma symptoms and growth (32). These findings are consistent with a growing body of research indicating that self-compassion can mitigate the emotional impact of trauma by reducing self-criticism and facilitating adaptive emotion regulation (26, 67). Evidence from non-disaster contexts further supports this interpretation: self-compassion has been inversely related to PTSD symptom severity (68), and interventions aimed at cultivating self-compassion (e.g., loving-kindness meditation) have been associated with reductions in trauma-related symptoms (69). While self-compassion is not a coping construct traditionally emphasized in disaster research and was assessed in only a small number of studies in the present review, the available evidence suggests that self-compassion may represent a promising, but still preliminary and exploratory, target for interventions aimed at fostering psychological growth and resilience following disasters, pending replication in independent samples (70). Further research is clearly needed to determine its generalizability and effectiveness across diverse survivor populations.

Finally, while much of the present discussion has focused on adaptive coping, it is important to acknowledge that maladaptive coping tendencies were also associated with poorer psychological outcomes. For example, one study found that a greater tendency toward social comparison was linked to higher levels of depression and anxiety and lower life satisfaction among earthquake survivors (39). Such findings illustrate how certain coping styles can inadvertently exacerbate distress; frequent comparison with others—particularly upward comparison—may intensify feelings of injustice, inadequacy, or frustration during recovery (71, 72). Other potentially maladaptive responses, such as denial, behavioral disengagement, or substance use, were noted anecdotally in some reports, although they were not examined quantitatively in the present synthesis. These observations underscore the dual nature of coping processes: coping strategies can facilitate recovery when adaptive, but may hinder adjustment when maladaptive or rigidly applied (73). Accordingly, disaster mental health interventions may benefit from approaches that both strengthen adaptive coping capacities and actively address maladaptive coping patterns, given consistent evidence linking avoidance-based strategies to the persistence of PTSD symptoms (74).

### Heterogeneity and moderators

A recurring feature of the present review was the variability (heterogeneity) in effect sizes across studies. Not all studies reported equally strong associations between a given coping strategy and psychological outcome, and statistical indices indicated substantial between-study heterogeneity for several pooled estimates. While specific cases have been discussed above (e.g., social support and positive reappraisal), several general factors may help explain this pattern.

One key source of heterogeneity is the timing of assessment. Included studies ranged from surveys conducted within the first 1–2 months following the earthquakes to assessments carried out nearly one year post-disaster. This temporal variation is important, as the psychological trajectory of disaster survivors is known to evolve over time (1). In the immediate aftermath, acute stress reactions and survival-related concerns often dominate, whereas in later phases, cognitive appraisal, meaning-making, and longer-term coping processes may become more salient (75). Accordingly, even individuals with high resilience may report substantial distress shortly after the event, potentially attenuating observed coping–outcome associations at early time points. In contrast, studies conducted several months later—or closer to one year post-disaster—more consistently identified associations between coping processes and psychological outcomes, including posttraumatic growth (e.g., self-compassion and PTG) (32). Such temporal variability likely contributed to heterogeneity in the pooled estimates.

Differences in sample characteristics and exposure contexts also warrant consideration. The reviewed studies included diverse survivor groups, ranging from young adults and university students [e.g., (31, 36)] to general community samples and individuals residing in temporary shelter settings after extensive loss (34). Variations in exposure severity, displacement, and material loss may reasonably lead to different baseline levels of distress and differential capacity for coping (76). For example, samples with moderate exposure (such as student populations) may exhibit weaker coping–outcome associations, whereas samples drawn from highly affected settings (e.g., tent cities) may show stronger effects, given greater stress to buffer and potentially more uniform reliance on communal or spiritual coping resources.

Cultural, regional, and measurement-related factors further contribute to variability. Although southeastern Türkiye is broadly characterized by collectivist and religious cultural norms (77), meaningful micro-level differences (e.g., urban versus rural context) may shape preferred coping strategies. In addition, coping constructs were operationalized differently across studies. For instance, resilience was measured as a dispositional trait in some studies and as a coping-related process in others [cf. (37, 38)], while social support was assessed as perceived availability, satisfaction, or active support-seeking (35). Such differences in measurement and conceptualization can generate variation in observed effect sizes even when the underlying constructs are theoretically related (19).

Despite these sources of heterogeneity, it is noteworthy that the direction of associations was largely consistent across studies, with adaptive coping strategies generally associated with lower psychological distress and maladaptive tendencies linked to poorer outcomes (see Table 3). This convergence in directionality suggests that heterogeneity primarily reflects differences in effect magnitude rather than contradictory findings. Nonetheless, caution is warranted in generalizing any single pooled estimate across all survivor groups or contexts (78). The observed variability underscores the need for future research to formally examine moderators—such as time since trauma, exposure severity, age, gender, and cultural context—using longitudinal designs and adequately powered interaction analyses. While the present review explored subgroup patterns descriptively (e.g., stronger effects in more severely affected samples), these observations remain provisional and should be tested more rigorously in future studies [cf. (33, 34)]. Finally, some degree of heterogeneity may also reflect random error and the limited number of studies contributing to certain pooled analyses; when only two or three studies are available, estimates of between-study variability are inherently less stable (79) (see Table 2). As the empirical literature on the 2023 earthquakes expands, future meta-analyses will be better positioned to delineate true sources of heterogeneity.

A further theoretical contribution of the present review concerns the identification of plausible *contextual mediators* that may shape the relationship between coping strategies and psychological outcomes following large-scale disasters. Factors such as access to safe and stable housing, severity of economic loss, and levels of social cohesion are likely to influence both the availability and effectiveness of coping processes. For example, individuals who remained in insecure housing conditions or experienced substantial financial loss may have had limited opportunities to benefit from adaptive coping strategies, regardless of personal resilience or support-seeking tendencies. Similarly, social cohesion at the community level—reflected in shared norms, trust, and collective problem-solving—may amplify the protective effects of individual coping strategies by embedding them within supportive social environments. The absence of such contextual factors may help explain the heterogeneity observed across studies, particularly for coping strategies such as positive reappraisal and religious coping. Although these mediators could not be examined directly in the present synthesis due to limitations of the primary studies, their consideration is essential for developing integrative models of disaster.

### Theoretical and practical implications

Taken together, the findings of this review reinforce and extend several theoretical frameworks in trauma psychology. First, they are broadly consistent with classic stress and coping theory (13), which posits that individuals’ coping responses play a central role in shaping psychological adjustment to stressful events. Across the included studies, survivors who reported greater use of adaptive coping strategies—such as problem-focused coping, positive appraisal, and support-seeking—tended to show more favorable psychological outcomes, whereas maladaptive coping tendencies were associated with greater distress [e.g., (31, 40)]. These patterns support the transactional model’s emphasis on coping processes as key determinants of post-trauma adjustment, beyond the objective severity of exposure.

Second, the findings have implications for cognitive and social models of PTSD. Contemporary frameworks, such as the cognitive model proposed by Ehlers and Clark (80), emphasize the role of post-trauma cognitive processing, emotion regulation, and social context in the maintenance or alleviation of symptoms. The observed associations between coping-related factors—particularly resilience, perceived social support, cognitive reappraisal, and emotion regulation difficulties—and trauma-related symptoms are broadly compatible with these models. Notably, evidence from individual studies indicating that difficulties in emotion regulation may mediate the relationship between maladaptive cognitions and PTSD-related symptoms [e.g., (34)] suggests that targeting cognitive–emotional processes may be especially relevant in post-disaster interventions. However, given the predominantly cross-sectional nature of the evidence, such interpretations should be viewed as theoretically informative rather than causal.

Third, the observation that certain coping processes—such as resilience, meaning-making, and self-compassion—were linked not only to lower distress but also to posttraumatic growth connects the present findings to theoretical models of growth following adversity (10). These models posit that adaptive cognitive and emotional processes can facilitate both recovery and positive psychological change. Evidence from individual studies in the current review suggests that meaning-centered coping and self-compassion may be particularly relevant for growth-related outcomes (32, 36). This pattern is consistent with the “Janus-face” perspective on trauma, which conceptualizes distress and growth as potentially co-occurring and influenced by overlapping coping mechanisms (81). At the same time, the limited number of studies assessing these constructs indicates that conclusions regarding growth-related processes remain exploratory and warrant further investigation.

From a practical standpoint, the findings highlight several considerations for disaster mental health support. Most broadly, they underscore the importance of incorporating psychosocial support and coping-focused approaches into post-disaster recovery efforts (82). While acute crisis management and treatment of severe psychopathology remain essential components of disaster response, the present evidence suggests that supporting survivors’ existing coping resources may also play a meaningful role in psychological adjustment.

In particular, community-based approaches that foster social support may be especially valuable. Facilitating peer support groups, strengthening family and community networks, and promoting opportunities for shared coping may help mitigate isolation and distress in disaster-affected populations (83). Given the prominent role of family and community ties in Türkiye, collaboration with local community leaders and outreach efforts aimed at vulnerable or isolated individuals may enhance the reach and acceptability of such interventions. These implications align with the consistent association observed between perceived social support and lower trauma-related distress in the reviewed studies [e.g., (31, 34)].

Similarly, resilience-oriented interventions may represent a promising avenue for post-disaster support. Programs that enhance problem-solving skills, emotion regulation, and adaptive stress management could help survivors regain a sense of control and efficacy (84). Evidence from other contexts suggests that resilience training can yield modest improvements in psychological outcomes (85), and the relatively strong and consistent associations observed between resilience and distress in the present review support the relevance of this focus. Nevertheless, intervention effectiveness in earthquake-affected populations remains to be established through longitudinal and experimental research.

Cultural context is another critical consideration. In Türkiye, religious and spiritual coping represents an important source of meaning and support for many individuals (53). The observed associations between religious coping and psychological outcomes—although heterogeneous—suggest that spiritually informed approaches may be acceptable and potentially beneficial for some survivors. Engaging faith-based organizations and spiritual leaders in psychosocial support efforts may help bridge gaps in service delivery and enhance cultural congruence (86). Practical applications could include collaboration with local religious figures trained in psychological first aid (87) or ensuring that disaster shelters accommodate spiritual practices. Importantly, such approaches should emphasize positive and supportive religious interpretations, rather than punitive or guilt-inducing narratives (59, 88).

Finally, interventions drawn from clinical psychology may consider incorporating elements that target cognitive reappraisal and self-compassion, particularly in later phases of recovery. Trauma-focused cognitive–behavioral interventions can be adapted to encourage adaptive meaning-making and reappraisal (80, 89), while compassion-focused and mindfulness-based approaches may help reduce self-criticism and facilitate emotional regulation (90, 91). Although evidence for self-compassion in disaster contexts remains limited, preliminary findings suggest that it may support psychological growth and well-being for some survivors (32). Overall, the present findings point toward the potential value of multicomponent, culturally informed psychosocial strategies that combine social support enhancement, coping skill development, and sensitivity to local meaning systems—while underscoring the need for rigorous evaluation of such approaches in future research.

### Limitations

This review has several limitations that should be considered when interpreting the findings. First, all included studies employed observational, cross-sectional designs, which preclude causal inference; it remains unclear whether coping strategies influenced psychological outcomes or whether distress levels shaped coping responses. Longitudinal research is therefore needed to clarify temporal relationships and to more rigorously test proposed mediation pathways.

Second, reliance on self-report measures introduces potential sources of bias, including common-method variance and dependence on subjective assessments of trauma exposure, coping, and psychological symptoms. Sampling bias is another important concern. Most studies relied on convenience sampling—often through online platforms—which likely underrepresented older adults, individuals with limited internet access, and some rural populations. Samples also tended to skew younger and female, limiting generalizability beyond the accessible Turkish adult population. In addition, the cultural specificity of the included studies constrains applicability to other contexts, and no eligible data were available for non-Turkish populations affected by the earthquakes, such as Syrian refugees.

Methodological heterogeneity across studies further limited synthesis. Coping constructs were operationalized in diverse ways, and relatively little attention was given to maladaptive coping strategies, restricting comprehensive comparison across domains. Meta-analytic estimates were based on a small number of studies per outcome (typically two to four), which reduces statistical power and the precision of pooled effect sizes and limits the stability of heterogeneity estimates. Although efforts were made to identify regional and grey literature, the possibility of missing unpublished or null findings cannot be excluded. Finally, relatively few studies adjusted for key confounders—such as prior trauma exposure, socioeconomic status, or pre-existing mental health conditions—which may have influenced the observed associations.

### Future research directions

To advance understanding of psychological recovery following the Kahramanmaraş earthquakes and similar disasters, future research should prioritize longitudinal designs that track survivors across multiple time points (e.g., 6–24 months post-disaster). Such studies are needed to clarify how coping strategies relate to trajectories of PTSD-related symptoms, depression, and posttraumatic growth over time, to determine whether early coping predicts later adjustment, and to examine the timing and sequencing of symptom change.

In addition, experimental and intervention-based research is essential. Future trials could evaluate the effectiveness of resilience-oriented programs, structured peer-support interventions, or culturally adapted psychosocial approaches that incorporate religious coping or self-compassion. These studies would move beyond correlational evidence to test causal mechanisms and inform evidence-based disaster mental health practices. Importantly, such interventions should be rigorously evaluated using randomized or quasi-experimental designs.

Broader population coverage also represents a key research priority. Future studies should include children, adolescents, older adults, first responders, and individuals with disabilities—groups that are often underrepresented yet may face distinct psychological challenges following disasters. In particular, the coping processes and protective factors that support youth recovery (e.g., family routines, school-based support, peer relationships) remain insufficiently explored in the post-earthquake context.

Greater attention should also be directed toward maladaptive coping strategies, such as avoidance, rumination, or substance use, which may contribute to the persistence of psychological distress. Future research should quantify the prevalence of these strategies and examine their long-term consequences, as well as their interaction with adaptive coping processes.

Finally, cross-cultural and comparative research could help distinguish coping–outcome associations that are broadly generalizable from those that are context-specific. Comparative studies involving survivors from different disaster settings may clarify the influence of sociocultural factors—such as the role of religiosity or community structure—on the effectiveness of particular coping strategies. As the evidence base grows, future meta-analyses that integrate findings from the Kahramanmaraş earthquakes with global disaster research will be better positioned to enhance generalizability and refine priorities for intervention and policy.

## Conclusion

This systematic review with meta-analytic synthesis provides a comprehensive overview of the psychological impact of the 2023 Kahramanmaraş earthquakes and the role of coping strategies in survivors’ adjustment. Across the included studies, adaptive coping processes—most notably resilience and perceived social support—were consistently associated with lower levels of trauma-related psychological distress, including PTSD-related symptoms, depression, and anxiety. Evidence for other coping strategies, such as religious coping, positive reappraisal, and self-compassion, was more variable and, in some cases, based on a limited number of studies, but generally suggested potential benefits for psychological adjustment and, in certain contexts, posttraumatic growth. Overall, the findings highlight that how individuals cope meaningfully influences post-disaster mental health, although the strength of associations varies across coping domains and populations.

At the same time, important methodological constraints—particularly the predominance of cross-sectional designs, reliance on self-report measures, and sampling biases—limit causal interpretation. The observed associations should therefore be viewed as indicative rather than definitive. Nevertheless, the convergence of findings across diverse samples and settings suggests that enhancing adaptive coping capacities represents a promising direction for culturally informed disaster mental health efforts.

In sum, this review underscores that coping processes are integral to psychological recovery following large-scale earthquakes. Interventions that strengthen resilience, promote social connectedness, and support culturally embedded and meaning-oriented coping practices may help mitigate long-term distress and facilitate recovery. Continued research employing longitudinal designs, diverse populations, and rigorously evaluated interventions will be essential to refine these insights and to inform effective mental health responses in future disaster contexts.

## Data Availability

The original contributions presented in the study are included in the article/supplementary material, further inquiries can be directed to the corresponding author.
